# How Does (*E*)-2-(Acetamidomethylene)succinate Bind to Its Hydrolase? From the Binding Process to the Final Result

**DOI:** 10.1371/journal.pone.0053811

**Published:** 2013-01-07

**Authors:** Ji-Long Zhang, Qing-Chuan Zheng, Zheng-Qiang Li, Hong-Xing Zhang

**Affiliations:** 1 State Key Laboratory of Theoretical and Computational Chemistry, Institute of Theoretical Chemistry, Jilin University, Changchun, Jilin, People's Republic of China; 2 Key Laboratory for Molecular Enzymology and Engineering of the Ministry of Education, Jilin University, Changchun, Jilin, People's Republic of China; Weizmann Institute of Science, Israel

## Abstract

The binding of (*E*)-2-(acetamidomethylene)succinate (*E*-2AMS) to *E*-2AMS hydrolase is crucial for biological function of the enzyme and the last step reaction of vitamin B_6_ biological degradation. In the present study, several molecular simulation methods, including molecular docking, conventional molecular dynamics (MD), steered MD (SMD), and free energy calculation methods, were properly integrated to investigate the detailed binding process of *E*-2AMS to its hydrolase and to assign the optimal enzyme-substrate complex conformation. It was demonstrated that the substrate binding conformation with *trans*-form amide bond is energetically preferred conformation, in which *E*-2AMS's pose not only ensures hydrogen bond formation of its amide oxygen atom with the vicinal oxyanion hole but also provides probability of the hydrophobic interaction between its methyl moiety and the related enzyme's hydrophobic cavity. Several key residues, Arg146, Arg167, Tyr168, Arg179, and Tyr259, orientate the *E*-2AMS's pose and stabilize its conformation in the active site via the hydrogen bond interaction with *E*-2AMS. Sequentially, the binding process of *E*-2AMS to *E*-2AMS hydrolase was studied by SMD simulation, which shows the surprising conformational reversal of *E*-2AMS. Several important intermediate structures and some significant residues were identified in the simulation. It is stressed that Arg146 and Arg167 are two pivotal residues responsible for the conformational reversal of *E*-2AMS in the binding or unbinding. Our research has shed light onto the full binding process of the substrate to *E*-2AMS hydrolase, which could provide more penetrating insight into the interaction of *E*-2AMS with the enzyme and would help in the further exploration on the catalysis mechanism.

## Introduction

Vitamin B_6_ belongs to pyridine derivate, which mainly refers to pyridoxine, pyridoxal, pyridoxamine and their phosphonate esterification form [1]. In the human body, vitamin B_6_ exists in the active forms of pyridoxal-6-phosphate (PLP). PLP plays a vital role as the cofactor of a large number of essential enzymes, which focus many important physiological processes, including macronutrient metabolism, neurotransmitter synthesis, histamine synthesis, hemoglobin synthesis and function, and gene expression [2], [3]. In these processes, PLP covalently binds to substrate and stabilizes carbanion intermediates adjacent to an amino group [3]. The biochemical reactions using PLP as a coenzyme predominantly involve reactions with amino acid, such as transamination, decarboxylation, racemization, and beta- or gamma-elimination or replacement [3]. The classic clinical syndrome for vitamin B_6_ deficiency is a seborrhoeic dermatitis-like eruption, atrophic glossitis with ulceration, angular cheilitis, conjunctibitis, intertrigo, and neurologic symptoms of somnolence, confusion, and neuropathy [4].

**Figure 1:**
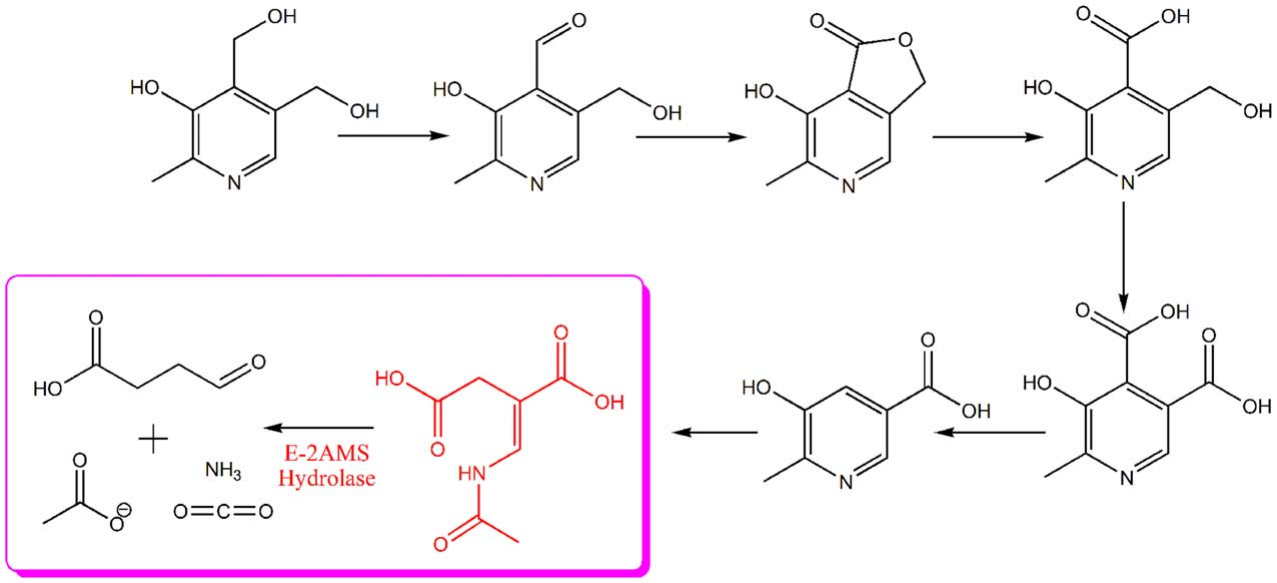
Vitamin B_6_ degradative pathway in *Mesorhizobium loti* MAFF303099.

For a long time, people have worked on biological functions and biosynthetic pathway of cofactor, whereas very little work has been reported on cofactor catabolism. Among all kinds of cofactors, the catabolism of vitamin B_6_ is the most studied and best understood cofactor catabolic pathway. In the late 1950s, vitamin B_6_ degradation was first discovered in the bacteria which grew on vitamin B_6_ as a sole source of carbon and nitrogen [5], [6]. Two catabolic routes for vitamin B_6_ degradation have been identified and characterized. In the first pathway, observed in *Pseudomonas* Sp. MA-1, vitamin B_6_ (pyridoxine) is degraded in eight steps to form succinic semialdehyde, while in the second catabolic pathway, found in *Pseudomonas* sp. IA and *Arthrobacter* Cr-7, it is catabolized in seven steps to 2-(hydroxymethyl)-4-oxobutanoate [6]. In the two degradative pathways, the involved catabolic enzymes and corresponding intermediates have also been identified and characterized [5]. Recently PLP catabolic genes has also been identified in a nitrogen-fixing symbiotic bacterium, *Mesorhizobium loti* MAFF303099, in which the catabolic route of vitamin B_6_ is similar to the first pathway seen in *Pseudomonas* Sp. MA-1 (Figure 1). All of the *M. loti* PLP catabolic genes have been identified and their encoded enzymes have also been well-characterized [7]–[15]. Crystal structures for four of these enzymes have been determined by X-ray crystallography [16]–[19].

(*E*)-2-(acetamidomethylene)succinate (*E*-2AMS) hydrolase is the last enzyme in the first vitamin B_6_ degradative pathway and it can hydrolyze *E*-2AMS to form succinic semialdehyde, ammonia, acetate, and carbon dioxide. The hydrolysis reaction has been well-characterized and the corresponding steady-state kinetic parameters have also been determined [13], [14]. Recently McCulloch and co-workers have proved that the hydrolase only utilizes *E*-2AMS rather than *Z*-2AMS as its substrate [13]. After that, they have also obtained the X-ray crystal structure of *E*-2AMS hydrolase encoded by the mlr6786 gene from *M. loti* MAFF303099 and identified several important residues involved in the substrate binding and catalysis reaction [19]. These studies have provided insight into the catalytic mechanism by *E*-2AMS hydrolase. Even so, the profound understanding of the hydrolysis reaction has still been incomplete by the failure in the cocrystallization experiments of the hydrolase with *E*-2AMS.

Molecular dynamics (MD) simulation is a powerful technique for investigating ligand-receptor interaction and binding process of ligand to enzyme [20]–[22]. The theoretical method can avoid many kinds of restrictions and difficulties in experiments, so it can provide a lot of useful information on the atomic level. However, until now, no theoretical studies have been reported on the enzyme selectivity, the enzyme-substrate interaction, and the binding process of substrate to enzyme. In the present work, several theoretical approaches were employed to investigate the selectivity of the hydrolase and the *E*-2AMS's binding to *E*-2AMS hydrolase-from the specific binding pathway to the final interaction mode. Based on the possible enzyme-substrate complex conformations obtained by molecular docking, conventional MD simulations and sequential binding free energies calculations were carried out to gain optimal enzyme-substrate complex conformation. Then substrate binding pathway was distinguished by steered molecular dynamics (SMD) simulations, and the potential of mean force (PMF) along the pathway was calculated on the basis of the SMD trajectories. The present simulations have provided us with insight into the enzyme selectivity and the binding of *E*-2AMS to its hydrolase and could be potentially used as a guide to further researches aimed at demystifying the hydrolysis mechanism of *E*-2AMS hydrolase and designing new hydrolase inhibitors.

## Results and Discussion

### Docking of *E*-2AMS and *Z*-2AMS into the Hydrolase

**Figure 2:**
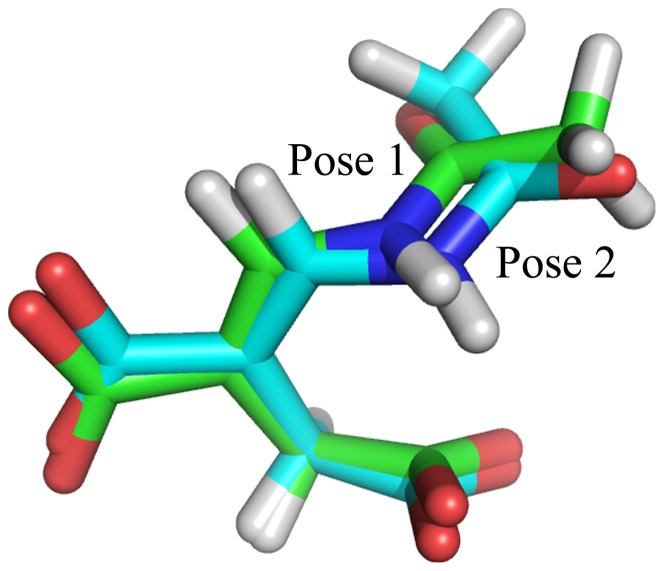
Two poses obtained from CDOCKER.

The experiment has determined the hydrolase's selectivity for *E*-2AMS over *Z*-2AMS [13], but the origin of such the selectivity is still unknown. And, up to now, the specific interaction between the hydrolase and its substrate has also been unclear as a result of the difficulty in obtaining X-ray structure of the enzyme-substrate complex. It precludes understanding of the *E*-2AMS hydrolysis mechanism. In our research, molecular docking was executed for generating the enzyme-substrate complex structures and illustrating the substrate selectivity of the hydrolase. According to the proposed reaction mechanism and the relevant reports [13], [14], [16], [19], the definition for the active site in molecular docking nearly covered all the known important residues, including the proposed nucleophilic residue Ser106, the catalytic residue His258, and their adjacent residues Arg146, Arg167 and Arg179. The docked results from CDOCKER presented 10 top poses of the substrates in the active site (Figure S1 and S2). Though careful inspection and comparison, it was found that these docked poses, for both *E*-2AMS and *Z*-2AMS, can be classified as two different binding modes. For the *Z* isomer, two different classes of docked poses have some similar interaction with the hydrolase (Figure S1). That is, in two classes of poses, the short carboxyl group of *Z*-2AMS both locates in the same site of the active pocket. (Here, the carboxyl group directly attaching to the double bond of 2AMS is temporarily named for the short carboxyl group, and the carboxyl group linking to the double bond by the methylene group is for the long carboxyl group.) It can be seen from Figure S1 that for either class of docked poses, the *Z* isomer always forms strong hydrogen bond (H-bond) interaction with the hydrolase's residues Tyr259, Arg146, Tyr168, and Arg167. Besides, in the 3 top docked poses, there also exist two H-bonds between Arg179 and the long carboxyl group (Figure S1(a)). It proves that these residues fundamentally affect the binding of *Z*-2AMS to the hydrolase. But whichever binding mode of *Z*-2AMS is disadvantageous to the catalysis of the substrate in view of the vacancy of the proposed oxyanion hole (consisting of Ile41 and Leu107) and the long distance of the nucleophilic residue Ser106 and the reactive carbon atom of the substrate. In contrast, for the *E* isomer, the 10 top docked poses, looking very similar, are all suitable for the substrate catalysis (Figure S2 and Figure 2). The important H-bond interaction mentioned above also appears in the docked poses of *E*-2AMS. Herein, by comparative analysis, the residues Tyr259, Arg146, Tyr168, Arg167 and Arg179 are the most important residues responsible for the proper orientation of substrate within the active site. On the basis, the different isomers show the different possibilities of reaction. So, the interaction between these residues and 2AMS results in the selectivity of the hydrolase for the *E* isomer.

For the 10 top docked poses of *E*-2AMS, there is still some slight difference in the configuration of the substrate. Figure 2 gives two representative conformations for the two classes of docked *E*-2AMS poses. It can be seen that two conformations merely differs in the amide bond parts, one of which has the *cis*-form configuration and the other is *trans*-form. (Here, in order to maintain consistency with the nomenclature of the *cis-trans* isomerism of the peptide bond in protein, we define Pose 1 as the *trans*-form configuration and Pose 2 as the *cis*-form configuration in Figure 2, respectively.) In the two poses, the amide hydrogen atoms both form the intramolecular H-bond with the nearest carboxyl oxygen atoms. The quantum chemistry calculations demonstrate that the configuration of *E*-2AMS with *trans*-form amide bond is lower 2.7 kcal mol^-1^ in energy than that with *cis*-form amide bond. Besides, the docked complex structures show that the amide oxygen atom of *trans*-form *E*-2AMS forms two H-bonds with the oxyanion-hole residues (Ile41 and Leu107) but the *cis*-form substrate does not have the similar H-bonds. Even so, it is still difficult to judge optimal binding conformation of the substrate in the hydrolase on account of the high degree of similarity between two configurations. Thus, long MD simulations for two kinds of binding conformations, accompanied by the calculations of binding free energy, were performed to verify the stability of *E*-2AMS in the protein and ascertain the optimal binding mode.

### Determination of the Optimal Binding Mode between *E*-2AMS and *E*-2AMS Hydrolase

Because of the initial structure of *E*-2AMS hydrolase with hydrogen atoms added by the external simulation program, there must be some unreasonable local region with high potential energy in the docked complex. Additionally, it needs to fully sample the conformational space of the complex in order to undertake the MM/GBSA binding energy calculation. Therefore, based on the molecular docking results of *E*-2AMS and the hydrolase, conventional MD simulations were carried out to obtain the more stable conformations for energy calculations as well as sequential SMD simulations. The structural stability of the hydrolase was assessed by the calculation of root-mean-square deviation (rmsd). The backbone's rmsd values of *E*-2AMS hydrolase for two substrate conformations are both generally stabilized near 1.0 Å in NPT simulations, favoring the equilibrium's accomplishment for two enzyme-substrate complex systems. The RMSD values of the active-site residues and the substrates also show the stability in the production simulations.

For the present system, the substrate's binding with *E*-2AMS hydrolase adopts two quite similar conformations, in which a lot of conditions are almost the same as each other. It is well known that the binding free energy from MM/GBSA methodology can provide semi-quantitative estimate for substrate's affinity with enzyme. Hence MM/GBSA energy can be used to identify the optimal binding mode of *E*-2AMS. Table 1 lists the binding free energies and their components for two substrate conformations. As shown in Table 1, it should be stressed that the binding free energies (Δ*G*_TOT_) of two interaction modes are both the negative values, indicating that they are energetically favorable. By comparing two binding free energies, it is obvious that Pose 1 with *trans*-form amide bond is lower in energy than Pose 2 with *cis*-form amide bond, suggesting that the substrate with *trans*-form amide bond is the highly probable binding conformation. For every component of the MM/GBSA binding free energies, the electrostatic energies (Δ*E*_ELE_) have much greater contribution to the total energies than the van der Waals energies (Δ*E*_VDW_). Hence it is worthwhile to mention that the electrostatic interaction is in the dominant position in the interaction of *E*-2AMS with the hydrolase. The total solvation energies (Δ*E*_GBSOL_), exactly the polar solvation energies (Δ*E*_GBCAL_), are disadvantageous to the binding of the substrate to *E*-2AMS hydrolase, albeit a minor contribution of the nonpolar solvation energies (Δ*E*_GBSUR_). The calculated results of solvation energies are consistent with the nature of the substrate, which features so many hydrophilic groups, resulting in the high energy-consumption of *E*-2AMS from the solution environment to the active site of *E*-2AMS hydrolase. Fortunately, such the energy consumption can be completely offset by the interaction between *E*-2AMS and its hydrolase, which eventually leads to the energetic favorableness of *E*-2AMS's binding to the hydrolase. From the detailed comparison of energy compositions, it can be concluded that the difference of two binding modes in the electrostatic energies (Δ*E*_ELE_) is the main reason leading to the advantage of Pose 1 in the final binding free energy, indicative of the importance of the electrostatic interaction. The inspection of simulation trajectories suggests that the non-stabilization of Pose 2 in the active site may result in the weakness of its interaction with the hydrolase. Of course, the contribution of entropy to the binding free energy, which can be derived from the last two columns of data in Table 1, further expands the advantage of Pose 1 in the free energy.

**Table 1:** Free energies and their compositions for two binding poses of *E*-2AMS to *E*-2AMS hydrolase (Unit:kcal mol^−1^).

	Δ*E*_ELE_	Δ*E*_VDW_	Δ*E*_INT_	Δ*E*_GAS_	Δ*E*_GBSUR_	Δ*E*_GBCAL_	Δ*E*_GBSOL_	Δ*E*_GBELE_	Δ*E*_GBTOT_	Δ*G*_TOT_
Pose 1	−284.67	−17.02	−0.00	−301.70	−3.34	264.80	261.46	−19.88	−40.24	−44.07
Pose 2	−261.80	−20.90	−0.00	−282.70	−3.41	255.25	251.84	−6.55	−30.86	−31.32

Δ*E*_ELE_  =  electrostatic energy, Δ*E*_VDW_  =  van der Waals energy, Δ*E*_INT_  =  internal energy,

Δ*E*_GAS_  =  interaction energy in gas phase (Δ*E*_ELE_ + Δ*E*_VDW_ + Δ*E*_INT_),

Δ*E*_GBSUR_  =  nonpolar solvation energy, Δ*E*_GBCAL_  =  polar solvation energy, Δ*E*_GBSOL_  =  total solvation energy (Δ*E*_GBSUR_ + Δ*E*_GBCAL_),

Δ*E*_GBELE_  =  total electrostatic energy (Δ*E*_ELE_ + Δ*E*_GBCAL_), Δ*E*_GBTOT_  =  enthalpy of binding (Δ*E*_GAS_ + Δ*E*_GBSOL_),

Δ*G*_TOT_  =  binding free energy.

From the calculations of MM/GBSA binding free energies, we can draw a conclusion that the binding conformation of the substrate with *trans*-form amide bond is energetically more favorable than the conformation bearing *cis*-form. In order to further validate the interaction of *E*-2AMS with the hydrolase on the atomic level, we has investigated the conformational change of the substrate in the active site during the 10 ns NPT simulations and taken statistics on the occurrence percentage of hydrogen bond between *E*-2AMS and the enzyme in the corresponding simulation stage. The NPT simulation trajectories imply that the substrate's pose with *trans*-form amide bond keeps synchronous movement with the hydrolase while the pose with *cis*-form amide bond is lack of such the synchronization. The *cis*-form substrate makes the relative rotation in the MD trajectories and can never keep the stabilization near some special conformation state in the simulation stage. The statistics results of hydrogen bonds show the more reliable interaction between the substrate and the enzyme. Table 2 represents the occurrence percentage of hydrogen bonds between two poses of *E*-2AMS and the related residues of the hydrolase in the whole NPT simulations. In this table, it is obviously noted that Pose 1 (*trans*-form) has more hydrogen bond interaction with the hydrolase than Pose 2 (*cis*-form), further certifying that *E*-2AMS with *trans*-form amide bond can more tightly bind to the enzyme. For Pose 2, some special attention needs be paid to the hydrogen bond with the residue His258, the occurrence percentage of which is high up to 28.3% in this pose of the substrate. However, in fact, the histidine residue is considered to be responsible for the activation of the nucleophilic residue Ser106 [19]. Remarkably, the formation of hydrogen bond between His258 and *E*-2AMS in Pose 2 must hinder the progress of the catalysis reaction. Consequently, Pose 2 with *cis*-form amide bond is not a reasonable binding conformation. Here, in order to estimate the consistency of the hydrogen bond statistics data, the present 10 ns MD trajectories were divided equally into five parts and taken respective statistics for every 2 ns trajectory. The results show that the occurrence percentage of hydrogen bond for Pose 1 maintains consistency between the global and local data, while that for Pose 2 has the considerable numerical fluctuation, which reconfirms the instability of Pose 2 in the hydrolase. Such the results are in satisfactory agreement with the observation in the NPT MD trajectories and further establish that the binding mode of *E*-2AMS bearing *trans*-form amide bond is the optimal enzyme-substrate complex conformation.

**Table 2:** H-bond's occurrence percentage between two *E*-2AMS poses and *E*-2AMS hydrolase's residues in NPT simulation.

Residue ID	Arg179	Tyr259	Arg146	Arg167	Ser106	Ile41	Tyr168	His258
Pose 1	96.50*	68.90	79.69	46.13	22.66	27.20	36.88	–
Pose 2	17.77	30.80	23.65	20.33	4.96	0.09	–	28.30

* All the numbers listed in the table are percentage.

### Interaction of the Substrate with *E*-2AMS Hydrolase

**Figure 3:**
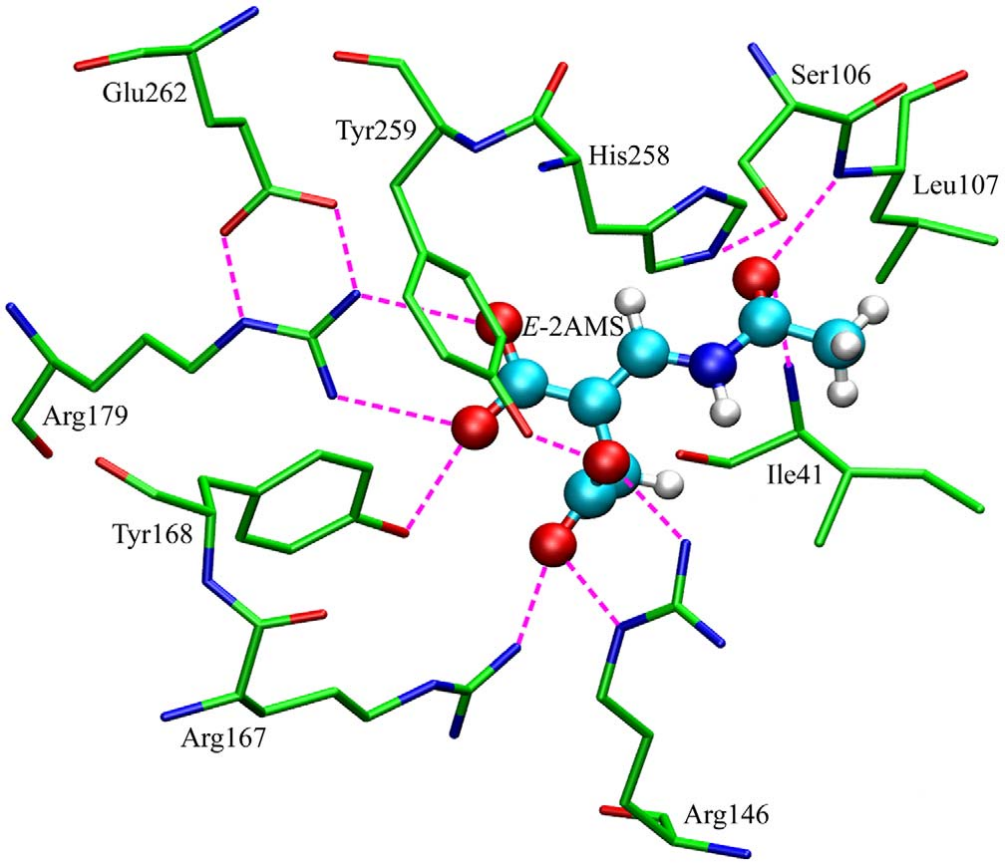
Interaction between *E*-2AMS and the key residues of *E*-2AMS hydrolase.

The detailed inspection of the atomic interaction between *E*-2AMS and the hydrolase was carried out on the basis of the determined optimal substrate binding mode. By the statistical analysis of the trajectory from the 10 ns NPT production simulation, we obtained the stable enzyme-substrate complex structure as presented in Figure 3. Most pivotal residues for the interaction of *E*-2AMS with the enzyme are also displayed. As shown in Figure 3, the substrate's binding to the hydrolase resorts to *trans*-form amide bond. Impressively, nine hydrogen bonds are formed between the substrate and the hydrolase, in which two carboxyl groups of *E*-2AMS forms seven hydrogen bonds with the hydrolase residues Arg146, Arg167, Tyr168, Arg179, and Tyr259. The additional two hydrogen bonds appear between the amide oxygen atom of *E*-2AMS and the main chain amide hydrogen atoms of the residues Ile41 and Leu107. The side chain hydroxyl oxygen atom of the nucleophilic residue Ser106, hydrogen bonded to His258, just locates over the *E*-2AMS amide carbon atom, which thus facilitates the trigger of the catalysis reaction. The hydrogen bond of *E*-2AMS with Ser106, whose occurrence percentage is up to 22.66% (Table 2), originates from the interaction between the hydroxyl hydrogen atom of Ser106 and the amide oxygen atom of the substrate. At the beginning of the hydrolysis reaction, this hydrogen bond will disappear in virtue of the deprotonation of Ser106 by His258. The presence of the hydrogen bond is the result of the present MD simulation not involving the initialization of the catalysis reaction. Actually, the occurrence percentage of the hydrogen bond between Ser106 and His258, up to 49.53%, is higher than the percentage between Ser106 and *E*-2AMS during the NPT simulation. Glu262 orientates and stabilizes Arg179 through two hydrogen bonds, confirming the interaction of Arg179 with the substrate.

It can be concluded from the analysis of the above-mentioned hydrogen bond interaction that when the catalysis reaction begins, the residues Arg146, Arg167, Tyr168, Arg179, and Try259 act the role of stabilizing the substrate in the active site. The residues Ile41 and Leu107, forming the hydrogen bonds with the amide oxygen atom of the substrate by their main chain amide hydrogen atoms, can offset the negative charge that builds up on the oxygen atom during catalysis, serving as an oxyanion hole, which fully coincides with the conclusion from the experimental structures [19], [23], [24]. Moreover, the present binding conformation with *trans*-form amide bond also provides probability and convenience for the interaction of *E*-2AMS with the oxyanion hole. Furthermore, this kind of conformation can also substantiate the formation of the hydrophobic interaction between the substrate's methyl group and the enzyme's hydrophobic cavity. (The cavity is not fully shown in Figure 3, mainly consisting of the residues Ile41, Leu107, Leu143, Leu207, Leu232, Ile135, Phe131, and Leu140.) For the conformation with *cis*-form amide bond, it not only hinders the interaction with the oxyanion hole, inducing the hydrogen bond with His258, but also destroys the hydrophobic interaction of the methyl group. It should be an important reason why the *cis*-form conformation can not keep stabilization during the MD simulation.

In the optimal binding conformation, all the hydrogen bonds are formed with the oxygen atoms of the substrate. Compared with the X-ray crystal structure of the free hydrolase, the locations of these oxygen atoms are originally taken up by water molecules. After the binding of *E*-2AMS, the oxygen atoms in the substrate take the place of these waters, binding to the enzyme. In this regard, the present binding conformation is also reasonable and reliable. And the present optimal enzyme-substrate complex conformation achieves remarkable consistency with the proposed complex structure by McCulloch et al. [19] Besides, no water molecules can stabilize near the active site during the NPT MD simulation. Concerning the interaction between *E*-2AMS and the catalytic residue Ser106, our simulation suggests that water molecule may be unnecessary in the initial stage of the hydrolysis reaction.

### Analysis of Binding Process of *E*-2AMS to the Hydrolase Based on SMD Simulations and PMF Calculations

**Figure 4:**
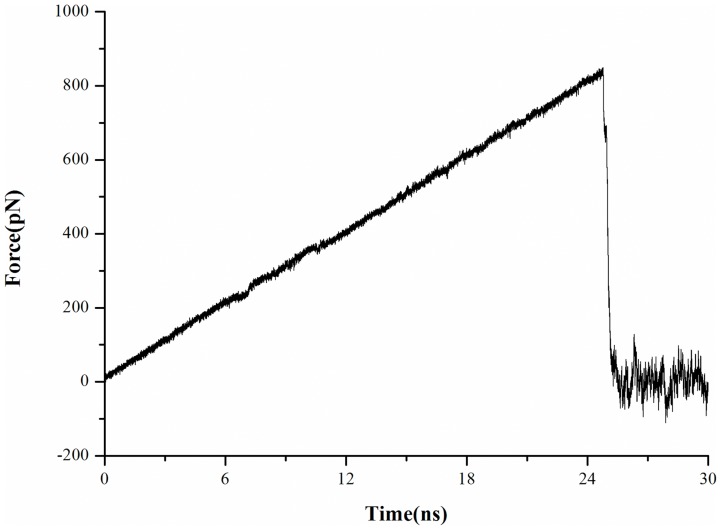
Time dependence of the external force for *E*-2AMS in the SMD simulation.

The binding or unbinding event of a substrate is difficult to reproduce in the time scales for conventional MD simulations which are generally confined to the order of tens of nanoseconds. Thus, in order to investigate the binding process of *E*-2AMS to its hydrolase, SMD simulations were further carried out based on the optimal complex conformation from conventional MD simulations. In the present research, cv-SMD simulation was performed to drive the dissociation of the substrate from the enzyme, which is the reverse process of binding. Figure 4 displays the changing profile of the external force *vs*. the simulation time. As shown in the figure, the pulling force exerted on *E*-2AMS remains increasing linearly in the initial stage of the SMD simulation. Afterward, at around 24.7 ns, the pulling force reaches to a peak value, which is just about 840 pN. At this moment, the substrate is about to leave the active site of the hydrolase. Then the value of the external force rapidly decreases to zero and features minor fluctuations near zero. Such the variation of the pulling force reflects the motion of the substrate in solvent, proving its full dissociation from the enzyme. On account of the structural character of *E*-2AMS hydrolase and the molecular size of the substrate, its unbinding pathway seems to be lack of some other possibilities. The attempts along the other different pulling directions display larger peak values of external forces. So the present chosen direction is regarded as the most probable binding or unbinding direction.

**Figure 5:**
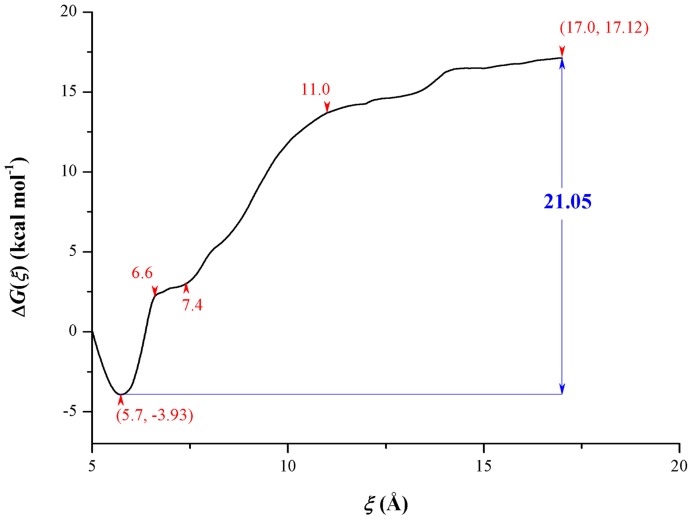
The PMF profile along the unbinding pathway of *E*-2AMS. As the reverse process, the free energy profile also applies to the binding process.

The investigation into the SMD simulation trajectories demonstrates that the substrate always stays in the active site until the external force increases to the maximum. After that, the substrate quickly leaves the hydrolase into the external solute environment. The unbinding process from the SMD simulation is so transient that some important conformation changes of the enzyme and the interaction with the substrate can not be timely observed in such the simulation. At the same time, with the purpose of calculating potential of mean force (PMF), the above-determined unbinding pathway, a 12 Å reaction coordinate, is artificially separated into twelve equal sections. For each of them, a fully sampling was carried out in up to 30 ns MD simulation. The calculated PMF profile is depicted in Figure 5. The free energy curve reveals some interesting information in the binding/unbinding pathway of the substrate. First, the minimum of energy in the profile is located at the site where the reaction coordinate is equal to 5.7 Å. In this point, the substrate *E*-2AMS is in the most stable binding state with the hydrolase. Less than the distance, the energy curve will sharply rise because of the repulsion between *E*-2AMS and the hydrolase. With the departure of the substrate from the equilibrium position, the free energy value rapidly increases, which is attributed to the interaction of the substrate with the active site residues. When the reaction coordinate is in the site of 6.6 Å, the enhancing trend of energy begins to slow down. After the point, until the site of 7.4 Å, the changing trend of free energy recovers the rapid rise and is kept for a longer distance. Beginning from the point of 11.0 Å, the energy curve displays the gentle increase. All the changes of the PMF profile intrinsically originate from the effect of the residues in the unbinding pathway. The interaction of the substrate with these residues will be in detail discussed below. An additional question worthy of note is that the free energy barrier of the substrate unbinding process is around 21.05 kcal mol^−1^ while the above-calculated value of the MM/GBSA binding free energy is −44.07 kcal mol^−1^. The discrepancy between two energies is about 23 kcal mol^−1^. On the one hand, the difference is ascribed to the different energy calculation methods; on the other hand two kinds of the calculation are not actually aiming at the exactly same process. The MM/GBSA calculation views the energy difference between the complex and fully dissociative states, while the PMF from the ABF method is the free energy change of the unbinding/binding process along the special pathway. In the profile shown in Figure 5, the end point of the curve corresponds to the state in which the substrate is on the surface of the hydrolase. At the moment, *E*-2AMS still has rather strong interaction with the enzyme.

**Figure 6:**
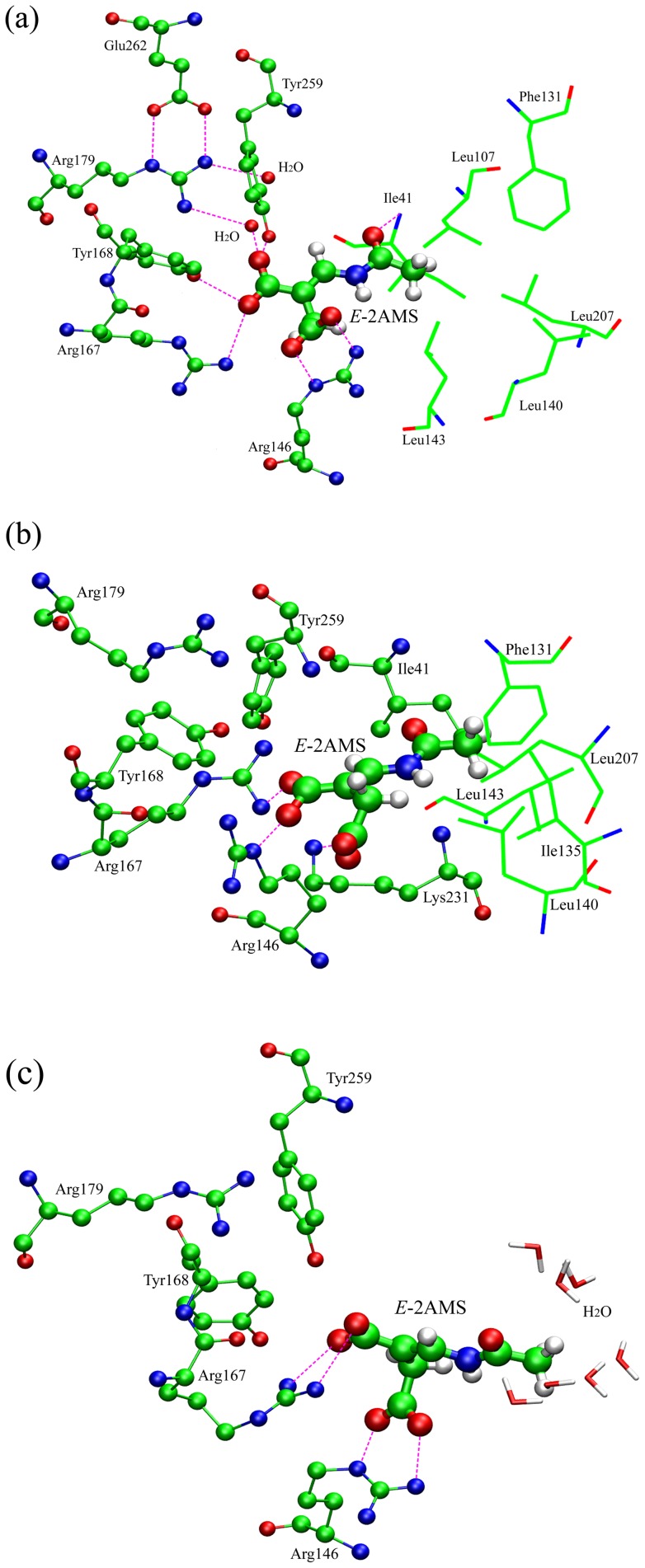
H-bond interaction of *E*-2AMS with the important residues of *E*-2AMS hydrolase in three key intermediates during the unbinding process of the substrate. The hydrophobic residues are shown in lines in (a) and (b).

The above PMF calculation reflects a great deal of important information on energy changes during the binding or unbinding process of the substrate, but it is still unknown which atomic interaction results in these energy changes. In order to explore the atomic essence underlying the energy changes, we carefully investigated the ABF simulation trajectories of the substrate's dissociation from the hydrolase. Notably, beginning with the most stable binding state (the reaction coordinate, *ξ*, is equal to 5.7 Å), the substrate *E*-2AMS gradually departs from the active site of the hydrolase. Inevitably, such the dissociation requires to get rid of the bondage of the active site residues. The observation of the trajectories shows that two hydrogen bonds between Arg179 and the short carboxyl group of the substrate are first broken, together with those two hydrogen bonds of the long carboxyl group with the residues Arg167 and Tyr259 (Figure 3). Arg167 and Tyr259 turn to the short carboxyl group of the substrate, forming two hydrogen bonds (Figure 6(a) and Table 3). Two external water molecules replace the oxygen atoms of the short carboxyl group to form two hydrogen bonds with the residue Arg179. Additionally, the hydrogen bond between the amide oxygen atom of the substrate and the main chain amide hydrogen atom of Leu107 also disappears as a result of the change of the substrate pose. Except several hydrogen bonds mentioned above, the other hydrogen bonds in the stable complex structure are still preserved. The methyl group of *E*-2AMS, with little change, still stays in the hydrophobic region of the enzyme. After the above changes, the obtained complex structure is a relatively stable intermediate conformation, shown in Figure 6(a), which is responsible for the slow growth of the PMF profile from 6.6 to 7.4 Å. Apart from the interaction presented in Figure 6(a), some water molecules also form hydrogen bonds with the substrate and some residues. Following the stable intermediate structure, the hydrogen bond breakage happens between *E*-2AMS and Ile41 (Figure 6(b)). The hydrogen bonds between the substrate and the residues Arg146 and Arg167 also become somewhat disorganized. The side chains of the two residues produce the bigger movement of conformational rotation, which opens up a channel for the unbinding of the substrate. Subsequently, two hydrogen bonds of *E*-2AMS with Tyr259 and Tyr168 are broken in succession. However, the hydrogen bonds still often appear between the short carboxyl group of *E*-2AMS and the residues Arg146 and Arg167. At the same time, the long carboxyl group of the substrate forms a new hydrogen bond with the residue Lys231 (Figure 6(b)). The breakage or formation of the above-mentioned hydrogen bonds finally leads to the rapid enhancement of free energy. Approaching the site where *ξ* equals 11.0 Å, the methyl group moiety begins to depart from the hydrophobic region of the active site, rotating to enter into the solvent environment. Such the adjustment of *E*-2AMS's pose contrarily brings about the consolidation of the hydrogen bonds between the substrate and the residues Arg146 and Arg167. The energy curve shows the slow growth. So it seems that *E*-2AMS's overcoming the hydrogen bond bondage of these related residues is the crucial reason why the free energy curve further rises between 13 and 14 Å of the reaction coordinate. From the above inspection and analysis, it can be observed that two residues Arg167 and Arg146, especially Arg146, always keep the hydrogen bond interaction with *E*-2AMS in the livelong unbinding process of the substrate (Figure 6 and Table 3). The statistics data of the hydrogen bonds along the reaction coordinate shows that the occurrence percentage of the hydrogen bond between Arg146 and *E*-2AMS exceeds 40% in the most case (Table 3). On account of always interacting with the substrate in the 12 Å long reaction coordinate, it is inferred that the side chains of two residues produce the larger conformation movement, thus facilitating the binding or unbinding of the substrate.

**Table 3:** The reaction coordinates dependence of the H-bond's occurrence percentage between *E*-2AMS and the hydrolase's important residues in the unbinding process.

*ξ*	Tyr 259	Arg 167	Ile 41	Arg 179	Tyr 168	Arg 146	Leu 107	Ser 106	Thr 42	Lys 231
5∼6	64.07^a^	27.71	23.36	117.76^c^	34.85	74.32	7.92	3.80	–	–
6∼7	47.16	45.34	15.90	15.26	40.94	87.98	2.78	35.72	0.64	–
7∼8	32.36	50.22	0.44	–	37.24	46.02	–	64.18	14.84	–
8∼9	8.72	20.62	–	–	56.92	22.76	–	1.14	–	3.90
9∼10	0.22	22.28	–	–	7.72	45.32	–	0.90	–	23.22
10∼11	0.38	27.78	–	–	0.78	33.28	–	0.88	–	10.58
11∼12	*–* b	42.48	–	–	6.32	44.88	–	–	–	5.44
12∼13	–	20.02	–	–	–	25.14	–	–	–	9.62
13∼14	–	0.02	–	–	–	42.04	–	–	–	1.70
14∼15	–	1.30	–	–	–	51.56	–	–	–	17.96
15∼16	–	43.16	–	–	–	44.10	–	–	–	2.42
16∼17	–	10.82	–	–	–	52.68	–	–	–	0.96

^*a*^All the numbers listed in the table are percentage.

^*b*^The sign stands for no values in the calculation.

^*c*^The value exceeding 100% originates from the coexistence of two hydrogen bonds between the substrate and the residue.

Besides the interaction of the substrate with the hydrolase's residues, we also did the further research on the interaction between the key residues of the enzyme. Arg179, responsible for the orientation of the substrate, is the first residue which needs to be paid attention to. In spite of the breakage of the hydrogen bond with the substrate, the residue Arg179 has almost no the conformational change in the *E*-2AMS's unbinding process, which is attributed to the stabilization role of the residue Glu262 (Figure 3 and 6(a)). The statistics data from all the 384 ns unbinding simulation gives the hydrogen bond occurrence percentage of up to 84.29% between two residues. For the important catalytic residues Asp130, Ser230, and His258, their hydrogen bond occurrence percentages are 62.79% for Asp130 and His258, and 74.61% for Asp130 and Ser230, respectively. Such the hydrogen bonds network ensures the stabilization of Asp130 by Ser230 and further the orientation of His258 through Asp130, which is the precondition for initialization of the catalysis reaction. The interaction between these important residues is well in line with the observation of the crystal structure [19].

**Figure 7:**
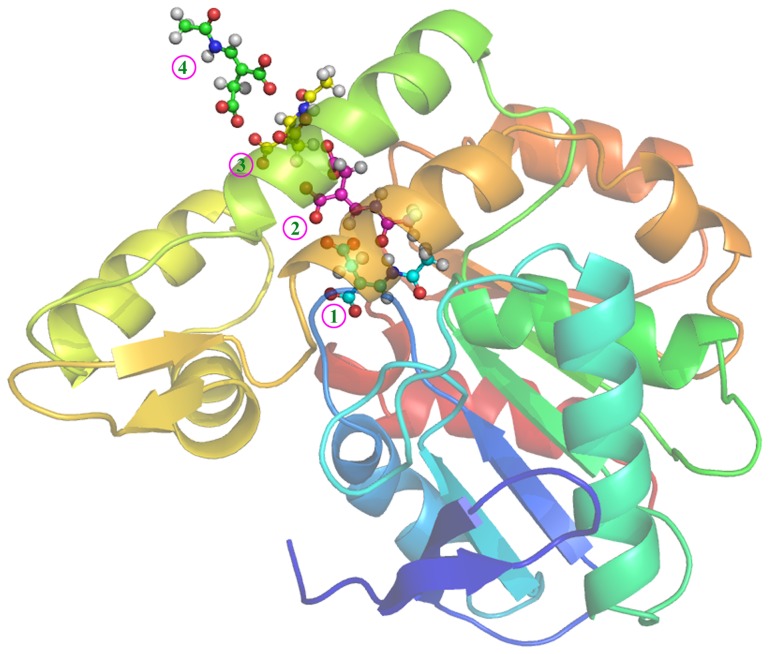
Snapshots of some key intermediate conformations during *E*-2AMS's dissociation from the hydrolase.

In the dissociation process, owing to the effect of the residues in the pathway, the substrate makes several necessary adjustments to its pose (Figure 7). Starting from the optimal binding conformation (Stage 1 in Figure 7), the carboxyl part of the substrate is the first to leave the active site, interacting with the adjacent residues and water molecules (Stage 2 in Figure 7). With the passage of simulation time, the hydrophobic part of the substrate also begins to depart from the active site. At this moment, the carboxyl parts of the substrate slowly move due to the attraction of the related residues (Arg146 and Arg167), whereas the methyl part quickly breaks away. The pose of the substrate resembles with the original stable state (Stage 3 in Figure 7). After that, the strong interaction of the residues with the carboxyl group of the substrate finally results in the first entrance of the methyl part into the water environment (Stage 4 in Figure 7). From Figure 7, it can be obviously seen that the interaction of *E*-2AMS's carboxyl groups with the related residues, specifically Arg146 and Arg167, is responsible for the pose change of the substrate in the unbinding process. The binding process of the substrate can also be distinctly presented by the investigation on such the reverse process of binding.

### Conclusions

Molecular docking of *E*-2AMS and *Z*-2AMS to the hydrolase suggests the importance of five key residues (Tyr259, Arg146, Tyr168, Arg167, and Arg179) to the hydrolase's selectivity. The detailed binding conformation of *E*-2AMS with its hydrolase was assayed by molecular docking and conventional MD simulations, followed by the binding free energy calculation. In the optimal enzyme-substrate complex structure, *E*-2AMS adopts the *trans*-form amide bond to interact with the related residues of *E*-2AMS hydrolase. The residues Arg179, Tyr168, Arg167, Tyr259, and Arg146 form seven hydrogen bonds with the substrate, greatly stabilizing its conformation in the active site. The *trans*-form amide structure of *E*-2AMS not only ensures the hydrogen bonds formation of its amide oxygen atom with the oxyanion hole (consisting of the main chain amide groups of Ile41 and Leu107) but also provides probability of the occurrence of the hydrophobic interaction between the substrate's methyl group and the enzyme's hydrophobic cavity. On the basis of the determined optimal complex conformation, the SMD simulation and PMF calculation were performed for the study on the binding process of the substrate. The obtained free energy profile for *E*-2AMS's dissociation reveals some crucial and interesting information on the interaction of the substrate with the hydrolase. Several key intermediate structures, together with some significant residues for the binding or unbinding of *E*-2AMS, have been discovered by the careful investigation into the long MD simulation trajectories. Among all the important residues, Arg146 and Arg167 almost always keep the hydrogen bond interaction with the substrate in the whole unbinding process. It is the interaction of the two residues with *E*-2AMS that is responsible for the conformational rotation of the substrate in the dissociation process. As the reverse process of unbinding, it is believed that the binding of the substrate to the hydrolase will also have the same intermediate structures and conformation changes with the unbinding. Our results could shed light on the binding of *E*-2AMS to its hydrolase.

## Materials and Methods

The molecular docking of two substrate isomers (*E*-2AMS and *Z*-2AMS) to the hydrolase was performed on a Dell Precision WorkStation T5400 with the CDOCKER [25] module in the Discovery Studio 2.5 software package [26]. The conventional MD simulations and calculations of binding free energies were carried out on the Inspur cluster with the Amber 10.0 package [27]. The SMD simulations and PMF calculations were performed with the parallel MD program NAMD 2.6 [28]. AMBER force field ff99SB [29] was assigned for *E*-2AMS hydrolase in both conventional MD and SMD simulations. The force field parameters for the substrate molecule *E*-2AMS were obtained from ab initio quantum chemistry calculations. All the geometric optimizations and energetic calculations of *E*-2AMS were implemented in the Gaussian 09 program package [30].

### Preparation of Initial Models

The X-ray crystal structure of *E*-2AMS hydrolase was obtained from the Protein Data Bank (www.rcsb.org), PDB code: 3KXP. In the crystal structure there are twelve chains of the hydrolase, water molecules, and several chloride ions. In our present research, chain A was chosen for molecular simulations, in which all the selenomethionine mutations were manually restored to methionine residues and the chloride ion was deleted.

The initial geometries of *E*-2AMS and *Z*-2AMS, with *cis*- or *trans*-form amide bond, were manually constructed by Discovery Studio 2.5 Visualizer [26]. Then the structural optimizations for these models and concomitant frequency calculations were employed by the density functional theory (DFT) with Becke's three-parameter (B3) [31] exchange functional along with the Lee-Yang-Parr (LYP) [32], [33] nonlocal correlation functional (B3LYP). The 6-31G(d) basis set was chosen, as implemented in Gaussian 09. The total charges and spin multiplicities for the quantum models were −2 and 1, respectively.

### Molecular Docking

The optimized geometries of *E*-2AMS and *Z*-2AMS with different amide bond were taken as the substrate's docking conformations. CDOCKER in the Discovery Studio 2.5 software package was used for their molecular docking into the hydrolase. CDOCKER is a grid-based molecular docking method that employs CHARMm [25]. The receptor was held rigid while the substrate was allowed to flex during structural refinement. Because the crystal structure of the hydrolase was obtained by the X-ray crystallography method, prior knowledge of its binding site had been acquired. Hence it was possible to specify the placement of substrate in the active site using a binding site sphere with the radius of 8 Å. Random ligand conformations were generated from the initial ligand structure through high temperature molecular dynamics at 1000 K, followed by random rotations. The random conformations were refined by grid-based simulated annealing and a final full force field minimization. The top 10 poses were saved for comparison and analysis.

### Conventional Molecular Dynamics Simulations and Calculation of MM/GBSA Binding Free Energy

The *E*-2AMS-hydrolase conformations obtained by molecular docking were utilized to perform conventional MD simulations. The whole system used Amber ff99SB force field. Because of the absence of the Amber force field for *E*-2AMS, we made use of quantum chemistry calculation to build the force field of the substrate. Based on *E*-2AMS's optimized geometries mentioned above, the HF/6-31G(d) basis set was employed to derive the partial charges of all the substrate's atoms, which was followed by RESP calculation in an antechamber module of Amber 10.0 package [27]. The additional three angle parameters for *E*-2AMS were obtained by analog from the generalized amber force field (GAFF) [34].

All the referred conventional MD simulations were carried out as the following procedure and methods. Hydrogen atoms were added with the XLeap module in Amber 10.0 package. Na^+^ ions were incorporated into the system in order to neutralize the negative charges. TIP3P [35] water molecules, filled in a truncated octahedral box extending up to 10 Å from the solute in each direction, were used to solvate the whole system. The final simulation system contained 32239 atoms. Periodic boundary conditions were applied to the system to obtain consistent behavior. The cutoff distance of van der Waals interactions were 1.2 nm. The particle mesh Ewald (PME) method [36], [37] was employed for the computation of electrostatic forces. An integration time step of 1 fs was assumed. The whole system was first energy minimized with 5000 steps of steepest descents and 5000 steps of conjugate gradients, by keeping the coordinates of protein and ligand atoms restrained with a spring constant of 500 kcal mol^−1^ Å^−2^. After minimization, at the same restraint, 1 ns MD simulation at 300 K was performed with the Langevin dynamics [38] method in the canonical (NVT) ensemble. Then 2 ns equilibrium simulation without any restraint was implemented to fully optimize the whole system. Following that, the pressure of the system was coupled to a reference pressure of 1 bar with a modified Nosé-Hoover Langevin piston method [39]. In the constant pressure/constant temperature (NPT) conditions, a 10 ns MD simulation was performed on the whole system to ensure the accomplishment of a production simulation.

The calculation of binding free energy was carried out by means of the MM/GBSA method. The method combines the molecular mechanical (MM) energies with the continuum solvent approaches by the programs in the Amber 10.0 package, in which the SANDER module is used for the calculation of the MM energies, the GBSA program [40] is used for the calculation of the polar solvent energy (the electrostatic contribution), and the MOLSURF program [41] is used for the calculation of the non-polar solvent energy (the non-polar contribution). In the present research, the energetic analyses for two binding conformations of *E*-2AMS both use the 500 snapshots from 10 ns of the trajectories at 20 ps intervals in their respective production simulations, together with the estimation of the solute entropic contribution by the NMODE module of Amber.

### Steered Molecular Dynamics Simulations and PMF Calculation

In order to investigate the binding process of *E*-2AMS to its hydrolase, SMD simulation was carried out to pull the substrate from the active site. SMD simulation is a computational approach for atomic force microscope (AFM), optical tweezers, biomembrane force probe, and surface force apparatus experiments. In SMD simulation time-dependent external forces are applied to a ligand to facilitate its unbinding from a protein. The analysis of the interactions of the dissociating ligand with the protein, as well as the recording (as a function of time) of applied forces and ligand position, yields important structural information about the structure-function relationships of the ligand-receptor complex and mechanism underlying the selectivity of enzymes. The method has been successfully used to study unbinding of ligands from proteins and has provided in-depth insights into the corresponding dissociation process [20]–[22]. In the NAMD implement, SMD may be carried out with either a constant force applied to an atom (or set of atoms) or by attaching a harmonic (spring-like) restraint to one or more atoms in the system and then varying either the stiffness of the restraint or the position of the restraint [28]. The two kinds of simulations correspond to constant force (cf) SMD and constant velocity (cv) SMD, respectively. In our system, the cv-SMD was adopted on the basis of the optimal complex structure from conventional MD simulation. A very small pulling velocity of 1 Å ns^−1^ and a force constant of 0.5 kcal mol^−1^ Å^−2^ were used, which should be appropriate to the present research on the unbinding process of *E*-2AMS according to our similar previous works [20]–[22]. While keeping the alpha-carbon (Cα) atoms of four residues (Ile41, Ser43, Arg167, and Arg179) fixed, external steering force was applied on the mass center of *E*-2AMS along the direction determined by the vector from the main chain N atom of residue Ile41 to the mass center of *E*-2AMS. The pulling force experienced by the reference point was calculated by employing Eq. 1:
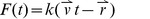
(Eq. 1)


where 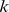 is the force constant for the spring and 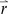 is the displacement of the reference point from its original position. During the SMD simulations, the steering force was only applied along the pulling direction. The substrate *E*-2AMS was free from constraint in the plane perpendicular to the pulling direction. A 2 fs time step, with the bonds containing hydrogen constrained by SHAKE, was used in the SMD simulations. The trajectory was saved for every 2 ps, and steering forces were recorded for every 1 ps. The overall 30 ns SMD simulation was performed to ensure the thorough dissociation of *E*-2AMS from the active site of the hydrolase. Then the obtained SMD trajectories were selectively extracted for PMF calculation.

To energetically investigate the dissociation of *E*-2AMS from its hydrolase, a reaction coordinate, *ξ*, was chosen as the distance between the mass centers of *E*-2AMS and the fixed atoms in SMD simulation. The potential of mean force, Δ*G*(*ξ*), along *ξ* was determined by the adaptive biasing force (ABF) method [42], which relies upon the integration of the average force acting on *ξ*. In the NAMD implementation of ABF [43], the force is evaluated within the classical thermodynamic integration formalism [44]. The free energy derivative, d*G*(*ξ*)/d*ξ*, is estimated locally throughout the simulations, thus providing a continuous update of the biasing force. Once applied to the system, the bias yields a Hamiltonian in which no net average force acts along *ξ*. As a result, all values of the reaction coordinate are sampled with the same probability, which, in turn, greatly improves the accuracy of the calculated free energies. To further increase the efficiency of the calculation, the dissociation pathway of *E*-2AMS–that is, 5≤ *ξ* ≤17 Å–was divided into 12 nonoverlapping windows. For each window, up to 30 ns of MD trajectory was generated. The initial Cartesian coordinates of the system for each window were gained from the above cv-SMD simulation. Instantaneous values of the force were accrued in bins 0.01 Å wide. Subsequently, an additional 24 ns ABF simulation was performed for the combination of free energy data from every separate run. The expression given by Rodriguez-Gomez et al. [45] was used to assess the standard error of the free energy difference.

## Supporting Information

Figure S1 **The interaction, with the hydrolase, of (a) the first 3 poses and (b) the rest 7 poses in the top 10 docked poses of** ***Z*** **-2AMS into the hydrolase.**
(TIF)

Figure S2 **Top 10 docked poses of** ***E*** **-2AMS into the hydrolase from CDOCKER.**
(TIF)
