# Supplementary figures and images for: How Does (E)-2-(Acetamidomethylene)succinate Bind to Its Hydrolase? From the Binding Process to the Final Result
Source: PLoS One. 2013 Jan 7;8(1):e53811. doi: 10.1371/journal.pone.0053811 (PMC3538738; doi:10.1371/journal.pone.0053811)

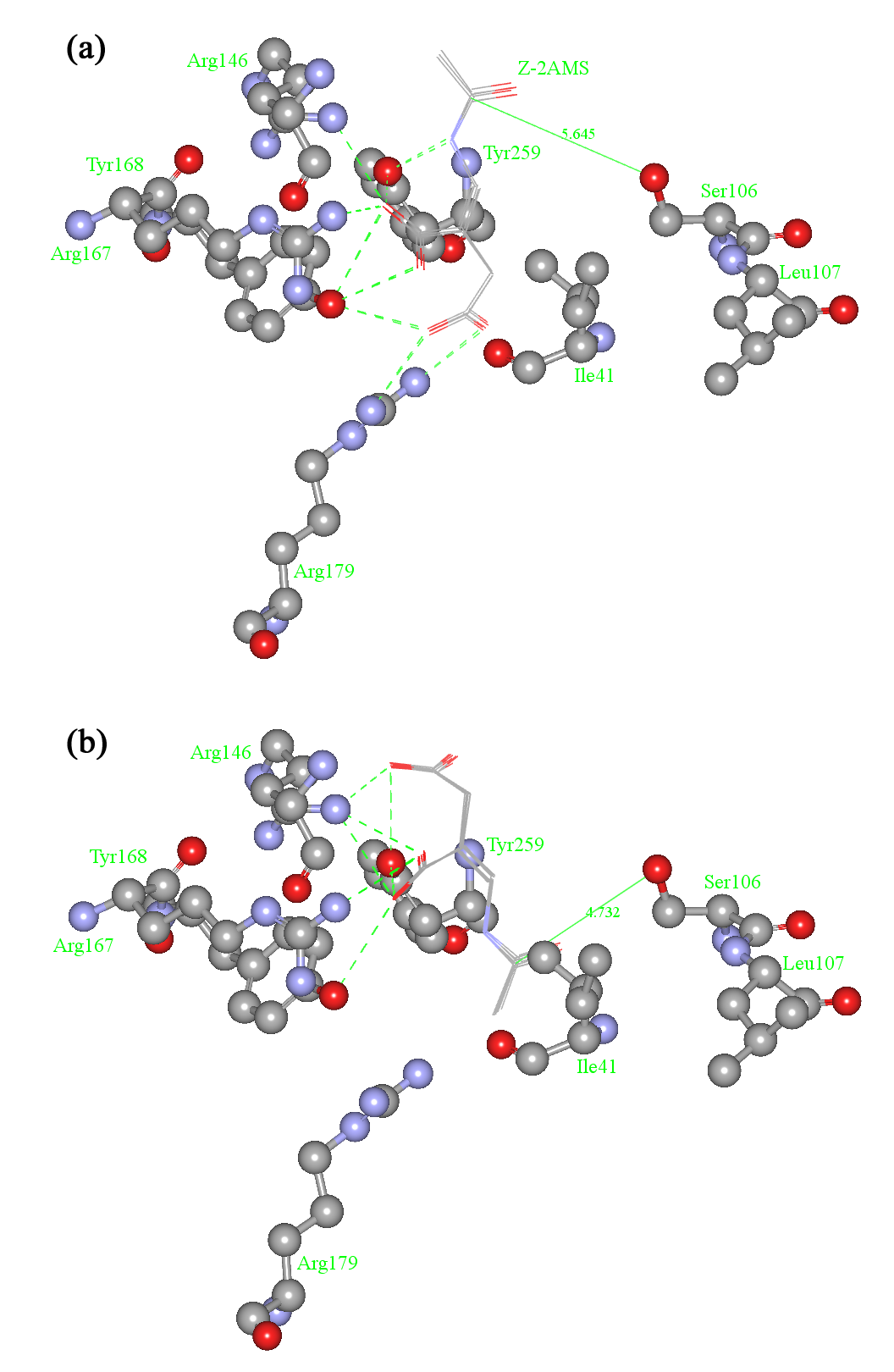

Supplement: Figure S1 — The interaction, with the hydrolase, of (a) the first 3 poses and (b) the rest 7 poses in the top 10 docked poses of Z -2AMS into the hydrolase. (TIF) [file pone.0053811.s001.tif]

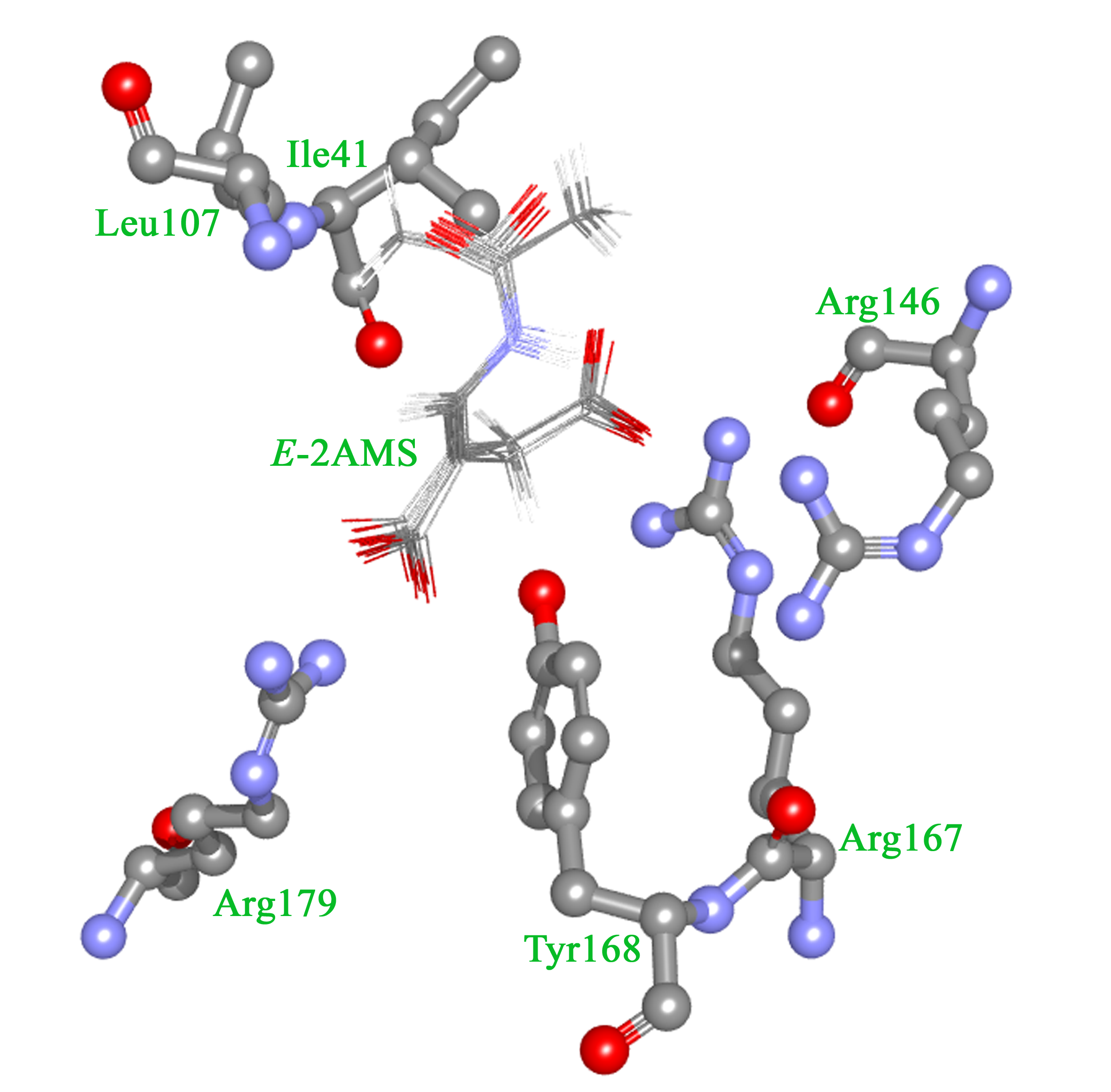

Supplement: Figure S2 — Top 10 docked poses of E -2AMS into the hydrolase from CDOCKER. (TIF) [file pone.0053811.s002.tif]
